# Iron and Nickel Mixed Oxides Derived From Ni^II^Fe^II^-PBA for Oxygen Evolution Electrocatalysis

**DOI:** 10.3389/fchem.2019.00539

**Published:** 2019-07-30

**Authors:** Zhuohong Xie, Chi Zhang, Xin He, Yi Liang, Dingding Meng, Jiaqi Wang, Ping Liang, Zhonghua Zhang

**Affiliations:** ^1^School of Applied Physics and Materials, Wuyi University, Jiangmen, China; ^2^Key Laboratory for Liquid-Solid Structural Evolution and Processing of Materials (Ministry of Education), School of Materials Science and Engineering, Shandong University, Jinan, China

**Keywords:** oxygen evolution reaction, oxygen vacancy, nickel ferrite, nickel oxide, Prussian blue analog

## Abstract

The sluggish kinetics of oxygen evolution reaction (OER) on anode hinders the efficiency of electrochemical water splitting. Electrocatalysts for OER based on non-precious transition metals are highly desirable. Herein, iron and nickel mixed oxides with surface oxygen vacancies were fabricated using Ni^II^Fe^II^-Prussian blue analog as the precursors by a facile two-step thermal-assisted method. The precursor compositions and calcination temperatures exert great impact on the structure and morphology of the derivatives, as well as the electrocatalytic performances for OER. Both the higher content of Ni ions during the synthesis of precursors and lower calcination temperature favor the electrocatalytic performance of the corresponding derivatives. The porous metal oxides consisting of nickel oxide and nickel ferrite exhibited the remarkable electrocatalytic property toward OER in an alkaline solution, which can be attributed to the nanosized and porous structure, the co-existence of spinel NiFe_2_O_4_ and cubic NiO, the high content of surface oxygen vacancies, and the low charge transfer resistance. This study will provide new inspiration for the facile design of low-cost active catalysts for OER in the future.

## Introduction

With the increasing concern over environmental protection and fossil fuel consumption, clean, and renewable energy like solar and wind power has attracted great attention. However, such energy is highly dependent on natural conditions, e.g., weather and region differences, and thus cannot guarantee the continuous energy supply. Water electrolysis is to convert the electric energy into hydrogen, supplying energy by combustion without the limitation of weather. Two thermodynamically uphill reactions are involved in the water electrolysis, i.e., hydrogen evolution reaction (HER) and oxygen evolution reaction (OER). With the endeavor of researchers, currently the overpotential to initiate HER has become quite low (Nørskov et al., 2005). However, OER on the anode of water electrolysis has more reaction barriers and larger overpotential is required, thus hinders the efficient production of hydrogen (Dau et al., 2010). Various electrocatalysts have been developed to boost the kinetics of OER. Ru and Ir-based oxides are commercially employed as the OER electrocatalysts, but the high cost and scarcity restrain the large-scale applications. Therefore, electrocatalysts based on the relatively cheap transition metals such as Fe, Ni, Mn, and Co are desirable to be explored.

Versatile transition metal catalysts, e.g., metal oxides, alloys, layered double hydroxides, oxyhydroxides, and phosphides have been studied for OER (Solmaz and Kardaş, 2009; Gong et al., 2013; Trotochaud et al., 2014; Xuan et al., 2017). Among them, Ni and Fe-based oxides were considered as the promising OER electrocatalysts. It is widely perceived that the Fe-doped nickel oxides outperform the cubic NiO and spinel Fe_3_O_4_ (Li and Selloni, 2014; Fominykh et al., 2015). Corrigan et al. discovered that Fe impurities had great effect on the OER catalytic performance of NiO, and a NiFe hydrous oxide with 10% Fe impurity showed enhanced electrocatalytic property compared with catalysts with less Fe or without Fe, requiring an overpotential of only ~200–250 mV to achieve the current density of 10 mA cm^−2^ (Corrigan, 1987). The nickel ferrite (spinel NiFe_2_O_4_) is a close-packed cubic oxide with Ni^2+^ occupying the tetrahedral holes and Fe^3+^ in the octahedral holes. The coexistence of Ni^2+^ and Fe^3+^ induces the hopping of bonding electrons, conferring NiFe_2_O_4_ with high electrical conductivity which is favorable for OER (Landon et al., 2012). It is also claimed that the crystalline metal oxides inclined to achieve better stability compared with the amorphous materials (Gong and Dai, 2015).

It was previously reported that the oxygen vacancies on the surface of the catalysts can improve the electronic conductivity and create more active sites, facilitating the electrochemical OER performance (Xu L. et al., 2016). Transition metal oxides with surface oxygen vacancies, such as FeCo oxide nanosheets, NiCo_2_O_4_ nanosheets, Co_3_O_4_ and MnO_2_, have showed enhanced electrocatalytic performance of oxygen reduction or evolution reactions (Cheng et al., 2013; Bao et al., 2015; Xu L. et al., 2016; Zhuang et al., 2017). The oxygen vacancies can create new defect states located in the bandgap of the metal oxides, where two electrons are easily excited, thus improving the conductivity of the metal oxides. Perovskite oxides with abundant oxygen vacancies are also generally applied as active OER electrocatalysts (Zhu et al., 2016). However, Ni and Fe-based oxides with oxygen vacancies for OER have rarely been reported previously.

Nickel and iron oxides with various morphologies, such as nanoparticles, films, hollow cubes, and nanofibers, have been prepared by spin-coating, aerosol spray, electrospinning, and microwave-assisted method (Trotochaud et al., 2012; Kuai et al., 2014; Li et al., 2015; Barforoush et al., 2017). Recently, metal organic frameworks (MOFs), consisting of metal nodes and organic linkers, have been widely used as the promising templates of carbon, metal oxides, and carbon-metal composites (Guo et al., 2015; Tang et al., 2015; Aijaz et al., 2016; Xu et al., 2017; Zheng et al., 2018). Compared with the conventional synthetic methods, templating from MOFs has the advantages of low cost, simple procedures and easily-adjustable compositions (Cao et al., 2017; Xie et al., 2017; Zou and Li, 2018). In addition, the derivatives can inherit the porosity of mother MOFs, which facilitates the mass transfer and exposure of active sites. For instance, Jiang et al. successfully synthesized Fe-Ni oxide architectures with different Ni and Fe contents as the electrocatalyst for OER, using Fe-Ni-based aminoterephthalate MOFs as the precursor (Jiang et al., 2016). Prussian blue analogs (PBAs) with the chemical formula A_x_M_y_[M′(CN)_6_]_z_·nH_2_O or M_y_[M′(CN)_6_]_z_·nH_2_O (A = alkali metal such as K and Na; M, M′ = transition metals like Co, Ni, and Fe) are frequently chosen as the MOF precursors to prepare transition metal-based materials (Kaneti et al., 2017; Li et al., 2018). Du et al. synthesized Zn-Fe-mixed oxide by thermal conversion of K_2_Zn_3_[Fe(CN)_6_]_2_ (Du et al., 2013). Han et al. achieved Ni-Co-mixed oxide by conversion of NiCo-PBA (Ni_3_[Co(CN)_6_]_2_) nanocages, manifesting enhanced OER activity (Han et al., 2016). Kang et al. prepared mesoporous Ni-Fe oxide hollow nanocage derived from Ni_3_[Fe(CN)_6_]_2_, showing a low overpotential and excellent durability in the electrocatalytic activities for OER (Kang et al., 2017). The studies above proved that PBAs could be excellent precursors to derive metal oxide-based electrocatalysts.

Inspired by this, we developed novel Ni-Fe oxides with surface oxygen vacancies as OER electrocatalysts derived from nanocrystalline Ni^II^Fe^II^-PBA (K_2_Ni_x_Fe_y_(CN)_6_) precursor. The synthesis involved two steps, including thermal-assisted synthesis of PBA precursors and pyrolysis conversion of PBA into metal oxides. The catalytic performance of the derived metal oxides was optimized by varying the calcination temperatures and Ni/Fe contents during synthesis. The results proved that the coexistence of NiFe_2_O_4_ and NiO facilitates the OER kinetics. Moreover, the surface oxygen vacancies were found to promote the electrocatalytic performance toward OER as well.

## Materials and Methods

### Materials

Polyvineypirrolydone (PVP) and isopropyl alcohol were purchased from Shanghai Macklin Biochemical Co., Ltd. Potassium ferrocyanide trihydrate (K_4_[Fe(CN)_6_]·3H_2_O) and nickel acetate tetrahydrate (Ni(CH_3_COO)_2_·4H_2_O) were obtained from Sinopharm Reagent Co., Ltd. Hydrochloric acid (HCl solution, 36%) and anhydrous ethanol were purchased from Guangdong Guanghua Sci-Tech Co., Ltd. Nafion solution (D520, 5 wt.%) from DuPont™ and Vulcan XC-72R carbon black from Cabot were used as received for the study. All reagents were used without further purification. Water (DI water, 18.2 MΩ) used for all the experiments in this study was purified through an Aquaplore 2S system.

### Synthesis of Nickel and Iron Oxides

**Figure 1:**
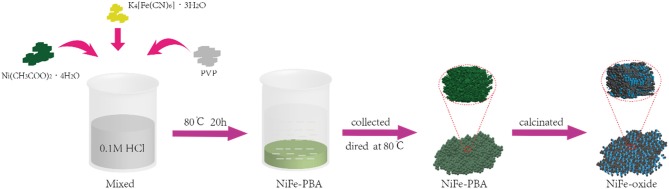
The flow chart of sample synthesis.

The synthesis routes of samples are illustrated in Figure 1. The PBAs with different Ni and Fe molar ratios were prepared as the precursors for metal oxides. The sample Fe-PB was synthesized without the addition of Ni(CH_3_COO)_2_·4H_2_O. First, 3 g of PVP and 0.4 mmol of K_4_Fe(CN)_6_·3H_2_O were dissolved in 0.1 M HCl (40 mL) and stirred rapidly for 30 min. Then the solution was placed in an oven for 20 h at 80°C. The blue precipitate was collected with centrifugation and washed thoroughly with DI water and ethanol several times, and finally dried in an oven at 80°C. The NiFe-based PBAs (including NiFe_2_-PB, NiFe-PB, Ni_2_Fe-PB, with the molar ratio of Ni ion and ferrocyanide at 0.5, 1.0, and 2.0, respectively) were prepared following the same procedures as above, except that Ni(CH_3_COO)_2_·4H_2_O was added. The NiFe-PBAs precipitates appeared pale green, due to the weak UV-vis absorption coefficient at around 520 nm as previously reported (Sato, 2006).

To fabricate the derived metal oxides, the as-synthesized PBAs were annealed in air at various temperatures. Specifically, the Fe-PB and NiFe-PBAs were transferred in the crucibles separately and calcinated in air at 500°C for 1 h with a ramping rate of 2°C min^−1^. The powders obtained were correspondingly named as Fe-O, NiFe_2_-O, NiFe-O, and Ni_2_Fe-O. In addition, the samples Fe-OX, NiFe_2_-OX, NiFe-OX, and Ni_2_Fe-OX (X: calcination temperature of 600 or 700°C) were also prepared. The sample names with the synthetic conditions are demonstrated in Figure S1.

### Characterizations

X-ray diffraction (XRD, X'pert Pro MFD) with a Cu Kα radiation (λ = 0.154178 nm) was used to investigate the phase compositions of the samples. The surface microstructure, morphology, and corresponding energy dispersive spectroscopy (EDS) spectra of the powders were obtained on a field emission scanning electron microscope (FESEM, Zeiss Sigma 500). The transmission electron microscopy (TEM), high-resolution transmission electron microscopy (HRTEM), selected area electron diffraction (SAED), scanning transmission electron microscopy-energy dispersive spectroscopy (STEM-EDS) mapping were obtained on a FEI Tecnai G2 F20 transmission electron microscopy. X-ray photoelectron spectroscopy (XPS, Thermo Scientific) was used to characterize the chemical states of the samples, using monochromatic Al Kα radiation. C 1 s electron binding energy at 284.6 eV was used for the correction of charge in all XPS spectra.

### Electrochemical Measurements

All electrochemical measurements were carried out in a three-electrode cell on a Solartron EnergyLab XM electrochemical workstation with a Model 636A Ring-Disk Electrode System at room temperature. A rotating disk electrode (RDE) with a glassy carbon disk (area of 0.196 cm^2^) served as the working electrode. The counter and reference electrodes were Pt plate and Ag/AgCl (saturated KCl-filled), respectively. All potentials measured were converted to the reversible hydrogen electrode (RHE) scale, according to E_RHE_ = E_Ag/AgCl_ + 0.059 pH + 0.197.

In a typical preparation procedure of catalyst ink, a mixture of 3 mg catalyst, 2 mg carbon black, and 15 μL Nafion was dispersed in 1.0 mL isopropyl alcohol solvent and ultrasonically treated for 30 min. Then, 10 μl of catalyst ink was pipetted onto the clean glassy carbon surface and dried in a vacuum tank to form a catalyst film, yielding a catalyst mass loading of 0.150 mg cm^−2^.

The OER polarization curves were carried out in an O_2_-saturated 1.0 M KOH electrolyte between 1.0244 and 2.0244 (vs. RHE) with a sweep rate of 5 mV s^−1^ at 1,600 rpm. The actual measurement was not initiated until a stable cyclic voltammetry (CV) curve appeared by running CV cycles repeatedly. All polarization curves were corrected with *iR*-compensation. Electrochemical impedance spectroscopy (EIS) for OER was conducted in an O_2_-saturated 1.0 M KOH electrolyte from 1,000 kHz to 0.1 Hz with an amplitude of 5 mV at 0.6 V vs. open circuit.

## Results and Discussions

**Figure 2:**
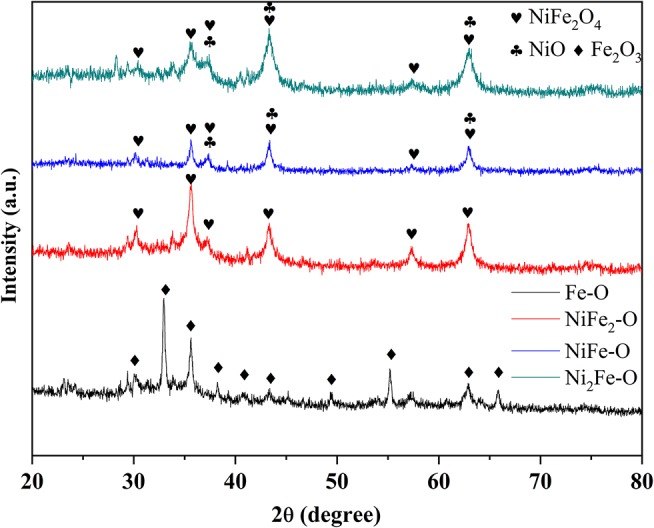
XRD patterns of samples annealed at 500°C.

The phase compositions of pristine Ni^II^Fe^II^-PBA were analyzed by XRD (Figure S2, Supporting Information). The diffraction peaks are independent of the Ni and Fe molar ratio, and all the patterns can be assigned to NiFe PBA with an NaCl-type structure as previously reported (Shigeyuki et al., 1997), indicating the successful synthesis of Ni^II^Fe^II^-PBA. No additional impurities were detected, suggesting the high purity of the products. The peak intensities of NiFe-based PBAs (including NiFe_2_-PB, NiFe-PB, and Ni_2_Fe-PB) became smaller compared with Fe-PB. It is possibly due to the addition of Ni ions, causing the distortion of the linear arrangement Fe-C-N-Ni in the NiFe-based PBAs (Hallmeier and Szargan, 2001). After calcination in air at 500°C, metal oxides formed as shown in Figure 2. Only Fe oxide (Fe_2_O_3_) was derived from Fe-PB while nickel ferrite (NiFe_2_O_4_) was obtained after pyrolysis of NiFe_2_-PB. The derivatives of NiFe-PB and Ni_2_Fe-PB were NiFe_2_O_4_ and NiO composites with clear phase separation. The intensity of diffraction peak at 43.8° (2θ) of NiFe-O was comparable to that of the peak at 35.6°, while the peak at 43.8° of Ni_2_Fe-O apparently had higher intensity than the peak at 35.6°, suggesting that more NiO exists in Ni_2_Fe-O than in NiFe-O. When the calcination temperature increased to 600 and 700° C, the same conclusions could be obtained from the XRD results of the derivatives (Figures S3, S4), indicating that the phase formation can be fully completed at 500°C, and phases of the derivatives are determined by the composition of the precursors, rather than the calcination conditions.

**Figure 3:**
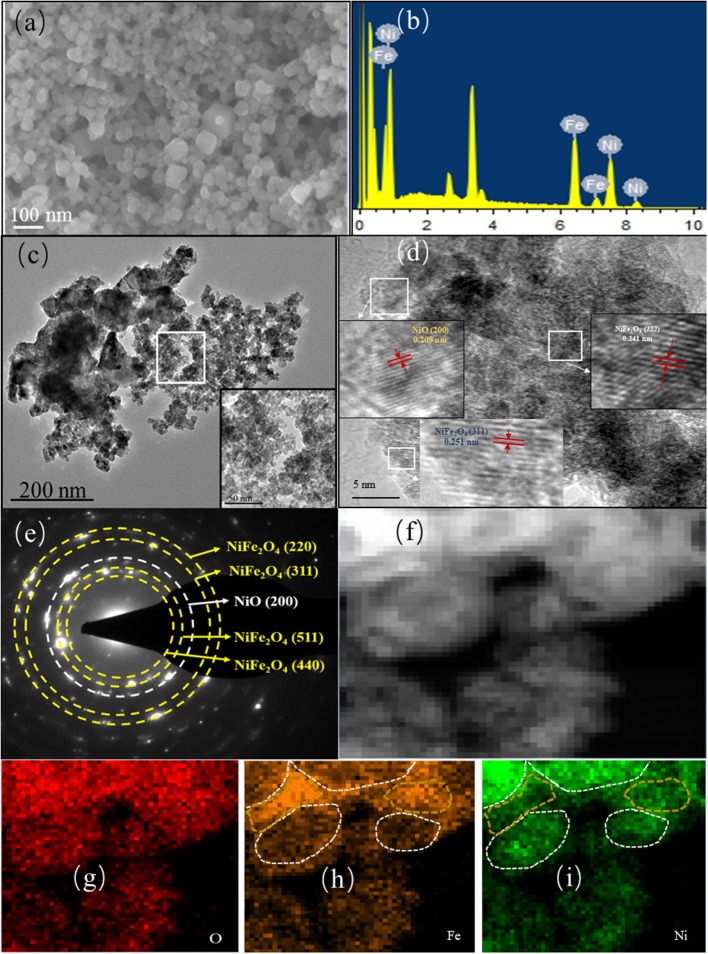
**(a)** SEM image, **(b)** EDS spectrum, **(c)** TEM image, **(d)** HRTEM image, **(e)** SAED pattern, and **(f–i)** element mapping of Ni_2_Fe-O.

Except for affecting the phase compositions, the introduction of Ni ions also influences the morphologies of the pristine PBAs. As shown in Figure S5, it displayed a cubic morphology on Fe-PB with the cubic size of ~400 nm. In contrast, the NiFe-based PBAs could not maintain the large cubic shape and consisted of ultrafine particles with the size of 20~150 nm. After calcination at 500°C, Fe-O inherited the large cubic shape of Fe-PB while nanosized cube-like particles were found on the rest Ni-Fe mixed oxides (Figure S6). As shown in Figure 3a, the particle size of Ni_2_Fe-O ranged from 20 to 150 nm. The particles with round corners distributed loosely with numerous pores located between. Particle aggregation could be observed when the calcination temperature increased to 600°C (Figure S7). When the calcination temperature rose to 700°C, the large cubic shape of Fe-O700 collapsed and particle aggregation accelerated on the Ni-Fe mixed oxides (Figure S8). The Ni and Fe signals could be found on the EDS spectra of NiFe-based PBA while only Fe peak existed on Fe-PB, indicating that the bimetallic PBAs are successfully synthesized (Figures S9a–d). Notably, the intensity of peak at 3.312 eV assigned to potassium on Ni_2_Fe-PB was lower than that on other three precursors. As previously reported (Shigeyuki et al., 1997), when the molar ratio of Ni ion to cyanoferrate ion increased to 1.5 during synthesis, insoluble cyanides containing no potassium ions took the priority to form instead of nickel-iron cyanide containing potassium ions. This could explain why less potassium content was discovered when the molar ratio of Ni and cyanoferrate ion was 2.0 in this study. As shown in Table S1, the content of Ni and Fe on the precursors mostly agreed with the molar design during the synthesis except Ni_2_Fe-PB. The molar ratio of Ni and Fe on Ni_2_Fe-PB was far below 2.0, because the insolubility of cyanides as discussed above hinders the precise control of composition during precipitation (Wessells et al., 2011). The element compositions and molar ratios of Fe and Ni of the derivatives after calcination at 500°C owned the same features as their corresponding precursors (Figures S9e–g, Figure 3b, and Table S2).

The TEM image in Figure 3c confirms the ultrafine Ni_2_Fe-O nanoparticles and porous structure, where darker parts present the particles and lighter areas denote the pores. The formation of the porous structure is attributed to the following two reasons: (1) the pores in the precursors were retained during the calcination; (2) the decomposition of linkers (-CN) in the precursor induced gas (such as CO_x_ and NO_x_) release during pyrolysis, creating pores in Ni_2_Fe-O. Figure 3d shows the HRTEM image of Ni_2_Fe-O, and the lattice spacing of 0.251 and 0.241 nm corresponds to the crystal planes (311) and (222) of the spinel NiFe_2_O_4_, respectively. In addition, a (200) planar spacing of 0.209 nm assigned to cubic NiO is also found. The corresponding SAED pattern (Figure 3e) reveals that Ni_2_Fe-O is polycrystalline and consists of NiFe_2_O_4_ and NiO, agreeing well with the XRD result. The STEM-EDS mapping profiles of Ni_2_Fe-O (Figures 3f–i) prove that Ni_2_Fe-O contains the elements Ni, Fe, and O, where Ni and Fe distribute non-uniformly. The areas in the gold circles are Fe-rich in the form of NiFe_2_O_4_ while Fe-rich parts in the white circles are mainly NiO.

**Figure 4:**
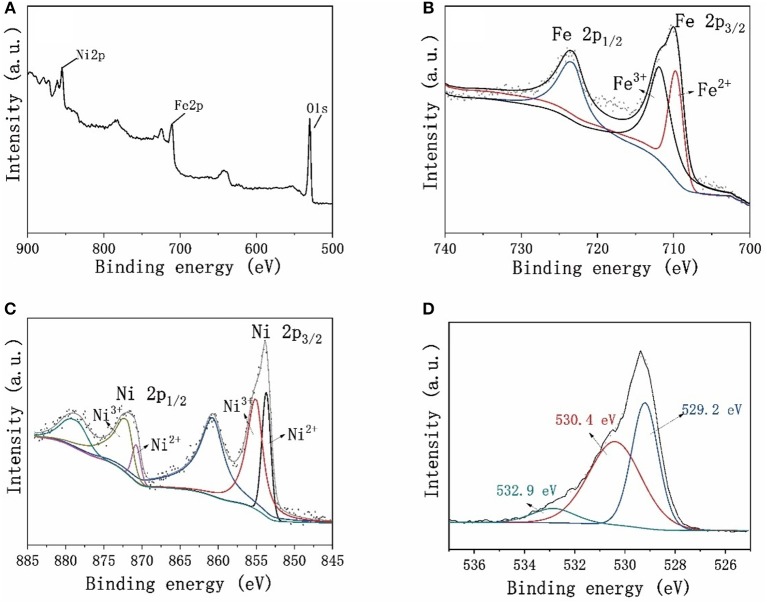
**(A)** The Full XPS survey spectra and the deconvoluted high-resolution XPS spectra of **(B)** Fe 2p, **(C)** Ni 2p, and **(D)** O 1 s obtained from Ni_2_Fe-O.

The surface chemical state of Ni_2_Fe-O was investigated by XPS. The full XPS survey spectra (Figure 4A) reveals that the surface of Ni_2_Fe-O includes Ni, Fe, and O. Figure 4B shows the Fe 2p doublet ranging from 700 to 740 eV. The binding energies at 710.3 and 723.8 eV are ascribed to Fe 2p_3/2_ and Fe 2p_1/2_, respectively, suggesting the formation of NiFe_2_O_4_ (Chen et al., 2010). The Ni 2p_1/2_ peak at 873 eV and Ni 2p_3/2_ peak at 854 eV are found in Figure 4C, and both peaks could be deconvoluted into components corresponding to the Ni^2+^ and Ni^3+^ oxidation states (Mcintyre and Cook, 1975; Biesinger et al., 2011). Moreover, the shake-up satellite peaks at 860 and 877 eV also reveals the Ni^2+^ and Ni^3+^ oxidation states (Uddin et al., 2015). The binding energy of O 1 s peak located at 529.2 eV (Figure 4D) is ascribed to bound oxygen in the lattice in the form of Ni-O and Fe-O (Solís et al., 2014). The fitting peak at 530.4 eV is the signal of the highly oxidative oxygen (${\text{O}}_{2}^{2-}$/O^−^) at the surface of Ni_2_Fe-O (Zhu et al., 2015; Xu X. et al., 2016). The component at 532.9 eV represents the adsorbed molecular water on the surface (Solís et al., 2014). Noteworthily, the oxygen in Ni_2_Fe-O is dominated by lattice oxygen and highly oxidative oxygen, accounting for 39.7 and 52.3%, respectively. As previously reported, the mixed Ni and Fe metal oxides are active components for OER (Gerken et al., 2014). Moreover, the highly oxidative oxygen representing the surface oxygen vacancies will lower the charge transfer resistance, and thus promote the OER activities (Gui et al., 2019). Thus, it is expected that the mixed phase composition and highly oxidative oxygen revealed by XPS results facilitate Ni_2_Fe-O to be a promising candidate for electrochemical OER.

**Figure 5:**
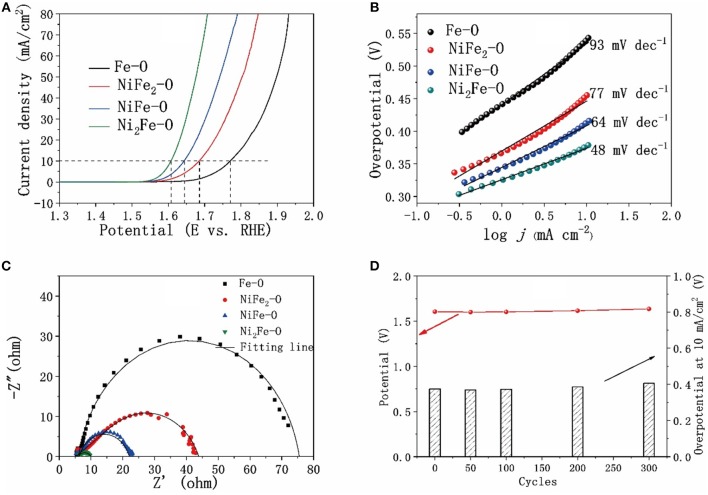
Comparison of electrocatalytic properties of derivatives (calcination temperature at 500°C) with different contents of nickel and iron. **(A)** OER polarization curves with *iR*-compensation, **(B)** Tafel plots of the derivative catalysts, **(C)** Nyquist plots of the catalysts, **(D)** Chronopotentiometric curve of Ni_2_Fe-O.

The catalytic activity toward OER of as-synthesized metal oxides was investigated. The *iR*-corrected linear scan voltammograms (LSV) curves in Figure 5A show that the electrocatalytic activities of the Fe-Ni mixed oxides were much better than the pure Fe_2_O_3_. Especially, Ni_2_Fe-O exhibited the best catalytic performance, requiring an overpotential (η^a^) of 370 mV to reach a current density of 10 mA cm^−2^ and an overpotential (η^b^) of 270 mV at the onset potential. The electrocatalytic activity of Ni_2_Fe-O in this study is also better than the previously reported results (Table S3) for NiFe_2_O_4_ nanoparticles (η^b^ = 470 mV) (Li et al., 2015), NiFe_2_O_4_ nanofibers (η^b^ = 440 mV) (Li et al., 2015), Fe_0.5_Ni_0.5_O_x_ (η^a^ = 584 mV) (Jiang et al., 2016), NiO (η^a^ = 430 mV) (Jung et al., 2016), NiFe_2_O_4_ (η^a^ = 500 mV) (Jung et al., 2016), Ni-Co mixed oxide porous cubes (η^a^ = 430 mV) (Han et al., 2016), and is comparable to the electrocatalytic performance of Fe_3_Ni_2_O (η^b^ = 270 mV) (Chen et al., 2014), NiOH nanoplate (η^a^ = 360 mV, η^b^ = 270 mV) (Yu et al., 2016), as well as Ni-Co mixed oxide cages (η^a^ = 380 mV) (Han et al., 2016). The catalytic kinetics of the catalysts are evaluated by the Tafel plot originated from the polarization curves. The lowest slope (48 mV dec^−1^) of Ni_2_Fe-O compared with the others suggests its favorable catalytic kinetics for OER (Figure 5B). The charge transfer resistance (R_ct_) of the catalysts exerts great influences on the catalytic performances and was investigated by electrochemical impedance spectroscopy (EIS), as shown in Figure 5C. The R_ct_ values of the catalysts are in good agreement with the corresponding electrocatalytic activity. The charge transfer resistance of Ni_2_Fe-O (~4.1 Ω) was the lowest, compared with that of NiFe-O, NiFe_2_-O, and Fe-O. To make it clear, the overpotentials at a current density of 10 mA cm^−2^, the Tafel slops and the R_ct_ values of the samples are summarized in Table S4. The stability of Ni_2_Fe-O was also studied as shown in Figure 5D. No apparent potential or overpotential rise was discovered in 300 cycles, suggesting the stability of Ni_2_Fe-O is reasonably well, benefiting from the high crystallinity. In particular, the overpotential happened to drop in the first 50 and 100 cycles with the corresponding R_ct_ decrease (Figure S10), which could be explained by that NiO undergoes an *in situ* transformation into the more active nickel hydroxide/oxydroxide for OER during the scanning (Trotochaud et al., 2012). This is also the reason why NiFe-O performed worse than Ni_2_Fe-O in the electrocatalytic OER here (Figure 5A). As discussed above by the XRD patterns, more NiO exists in Ni_2_Fe-O than in NiFe-O, resulting in more nickel hydroxide/oxydroxide forming during catalysis.

**Figure 6:**
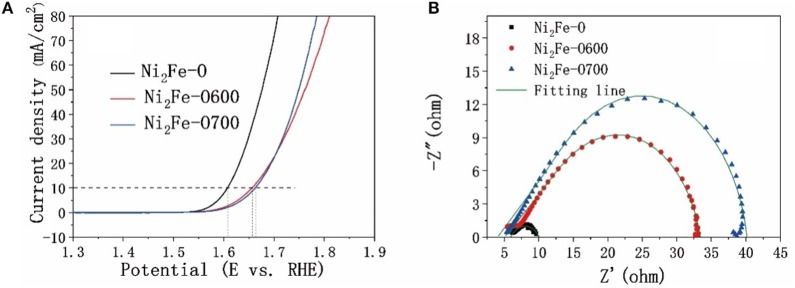
Comparison of electrocatalytic properties of derivatives under different calcination temperatures. **(A)** OER polarization curves with *iR*-compensation, **(B)** Nyquist plots of the catalysts.

In addition to the molar ratios of Fe and Ni during synthesis, the calcination temperature also has great impact on the electrocatalytic performance of the derivatives. As shown in Figure 6A, the Ni_2_Fe-O performed much better than Ni_2_Fe-O600 and Ni_2_Fe-O700. The charge transfer resistances of the samples are consistent with their electrocatalytic properties. The worse catalytic effect and higher R_ct_ values of Ni_2_Fe-O600 and Ni_2_Fe-O700 (Figure 6B) are attributed to the aggregation of nanoparticles as discussed above (Figures S7, S8), which hinders the mass transfer and reduces the exposure of active sites.

Based on the analysis above, the best electrocatalytic performance of Ni_2_Fe-O for OER in this study is attributed to the following reasons: first, the nanosized particles and porous structures facilitate the exposure of active sites, the diffusion of the electrolyte and enlarge the contact area of electrode and electrolyte (Yu et al., 2016); second, the spinel NiFe_2_O_4_ with high electrical conductivity is more active for OER (Li and Selloni, 2014) and cubic NiO can be transformed into active nickel hydroxide/oxydroxide during cycling; third, the high content of surface oxygen vacancies confirmed by XPS spectra contributes to the electrocatalysis (Zhu et al., 2016); the last, the smallest charge transfer resistance of Ni_2_Fe-O favors the catalytic activity for OER.

## Conclusions

Iron and nickel oxides derived from Ni^II^Fe^II^-PBA have been successfully synthesized by a facile two-step route. The molar ratio of Ni and Fe and calcination temperature during the synthesis have great influences on the morphology and structure of the derivatives, as well as the OER activity. The sample Ni_2_Fe-O consisting of NiO and NiFe_2_O_4_ nanoparticles exhibited the best among the catalysts in this study, requiring an overpotential of 370 mV to achieve a current density of 10 mA cm^−2^. The constituents, porous structure, surface oxygen vacancies, and low charge transfer resistance of Ni_2_Fe-O favor the electrocatalytic activity. This study opens a new perspective for the development of active catalysts for OER.

## Data Availability

All datasets generated for this study are included in the manuscript/Supplementary Files.

## Author Contributions

CZ and PL conceived and designed the experiments. ZX, YL, DM, and JW performed the experiments. XH, CZ, and ZX analyzed the data. ZX and CZ wrote the paper. PL and ZZ revised the paper.

### Conflict of Interest Statement

The authors declare that the research was conducted in the absence of any commercial or financial relationships that could be construed as a potential conflict of interest.
