# Supplementary material for: Iron and Nickel Mixed Oxides Derived From NiIIFeII-PBA for Oxygen Evolution Electrocatalysis
Source: Front Chem. 2019 Jul 30;7:539. doi: 10.3389/fchem.2019.00539 (PMC6689985; doi:10.3389/fchem.2019.00539)
Supplement: Supplementary file 1 [file Data_Sheet_1.pdf]

Supplementary Material

1 Supplementary Figures and Tables

1.1 Supplementary Figures

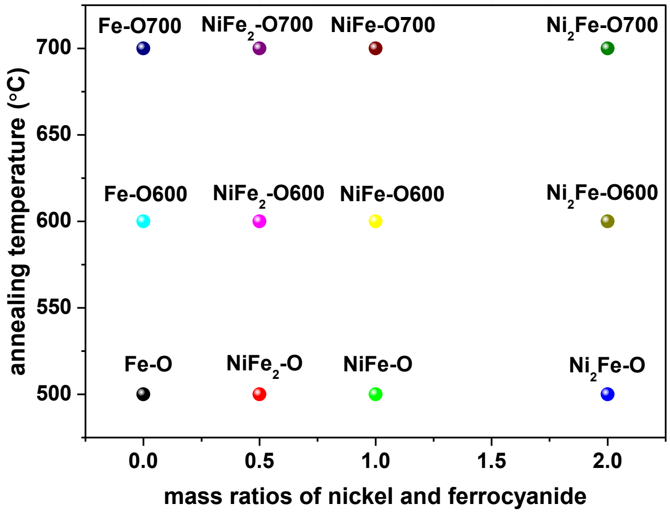

Figure S1. Sample names, the molar ratios of nickel and ferrocyanide, and annealing temperatures.

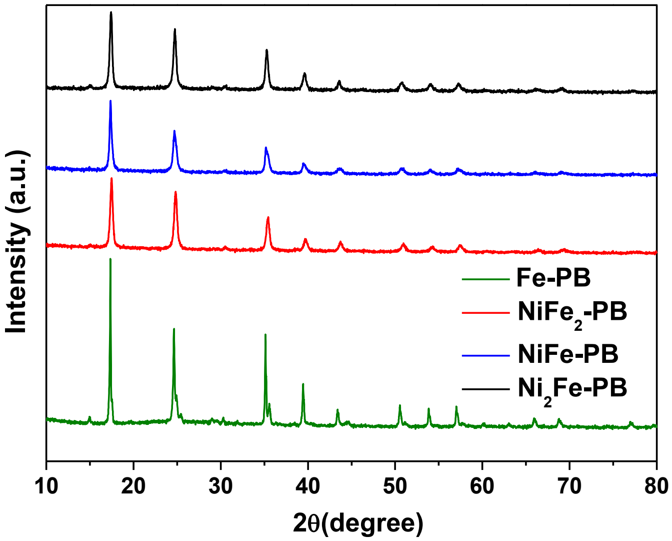

Figure S2. XRD patterns of pristine Prussian blue and analogues.

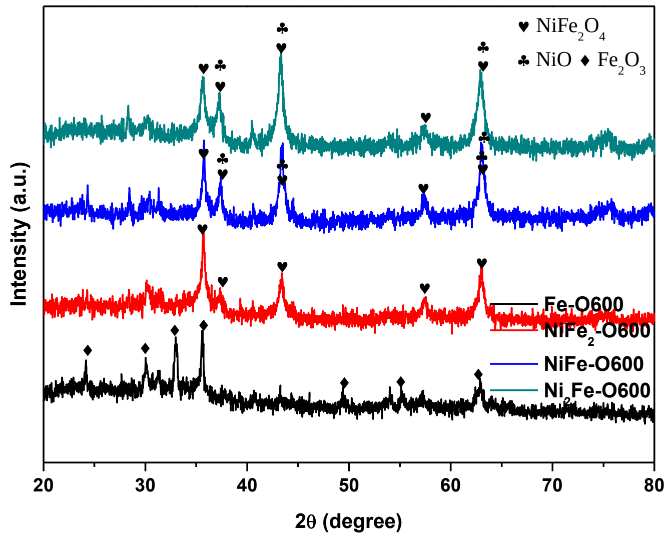

Figure S3. XRD of samples annealed at 600°C.

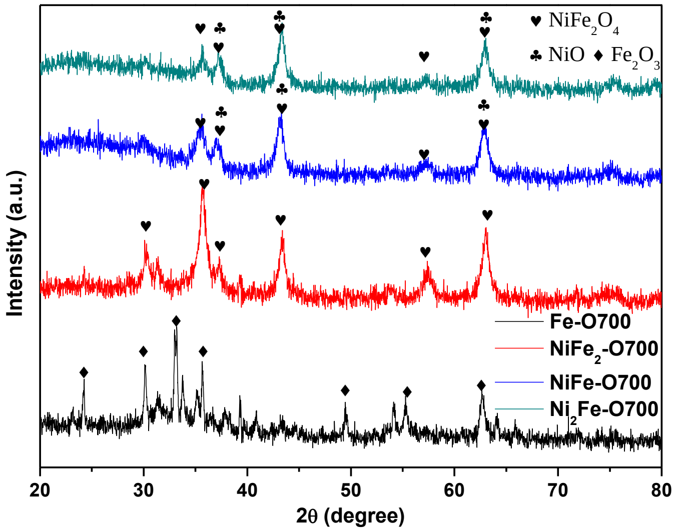

Figure S4. XRD of samples annealed at 700°C.

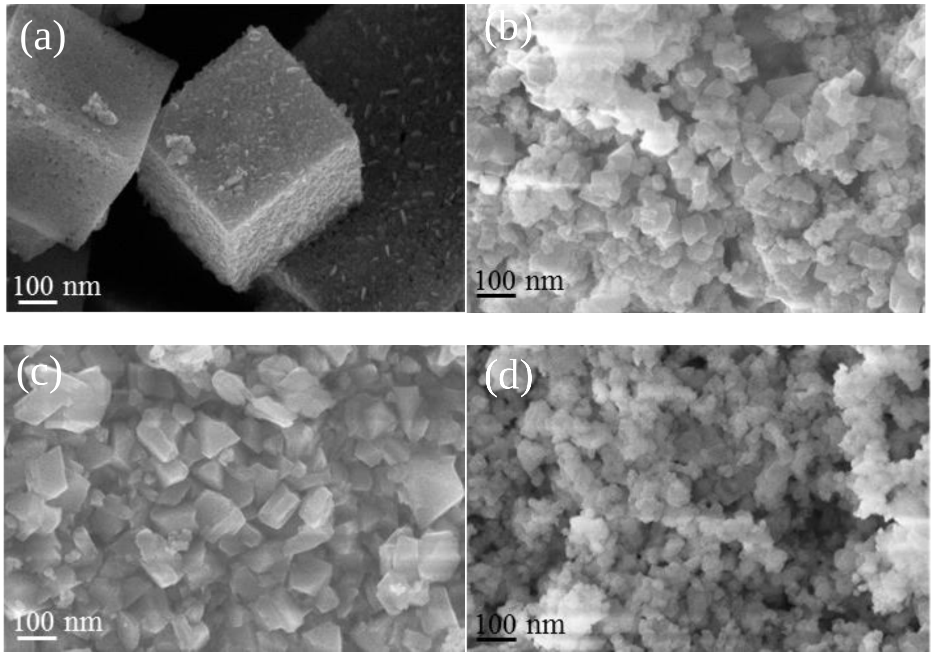

Figure S5. SEM images of Fe-PB (a), $NiFe_2$-PB (b), NiFe-PB (c) and Ni_2Fe-PB (d).

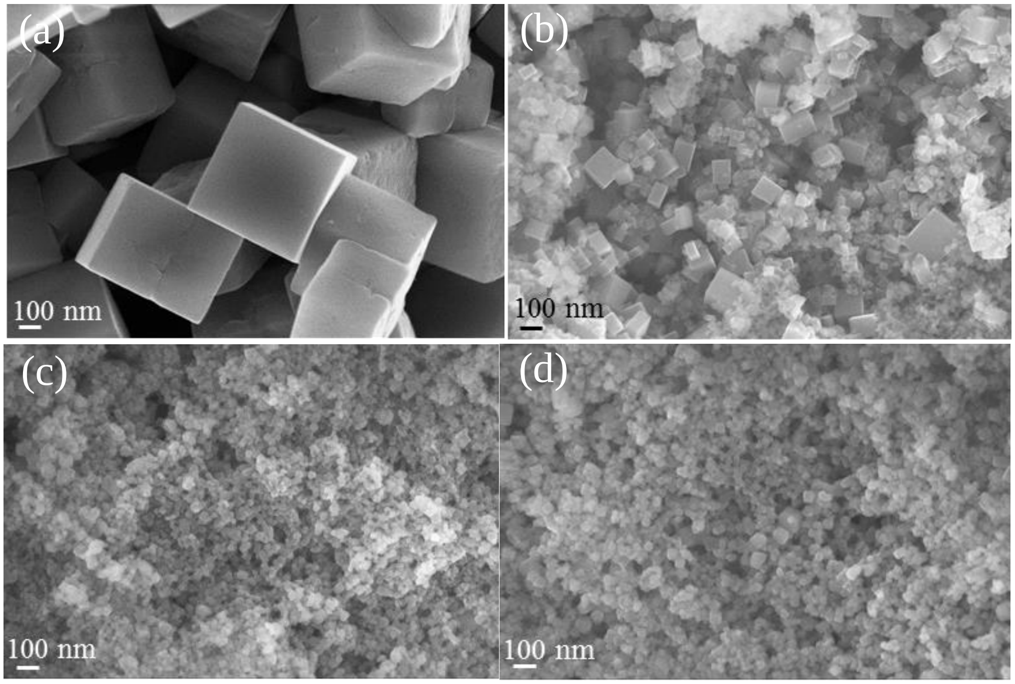

Figure S6. SEM images of Fe-O (a), $NiFe_2$-O (b), NiFe-O (c) and Ni_2Fe-O (d) after calcination at 500 °C.

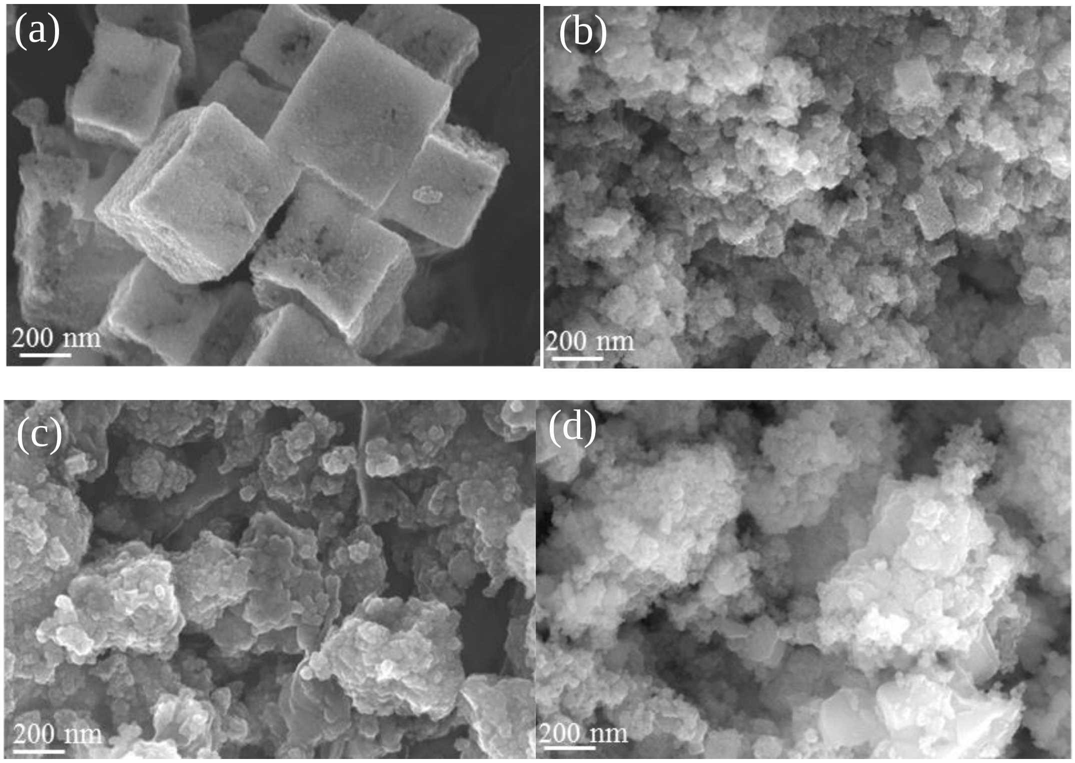

Figure S7. SEM images of Fe-O600 (a), $NiFe_2$-O600 (b), NiFe-O600 (c) and Ni_2Fe-O600 (d).

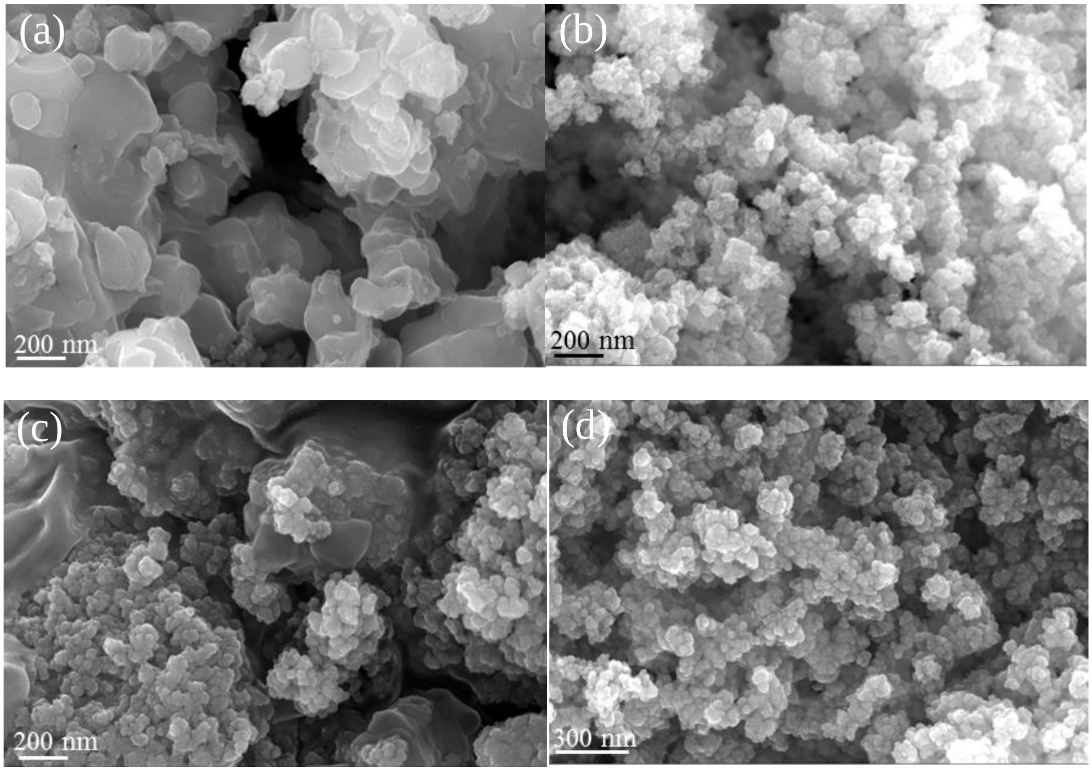

Figure S8. SEM images of Fe-O700 (a), $NiFe_2$-O700 (b), NiFe-O700 (c) and Ni_2Fe-O700 (d).

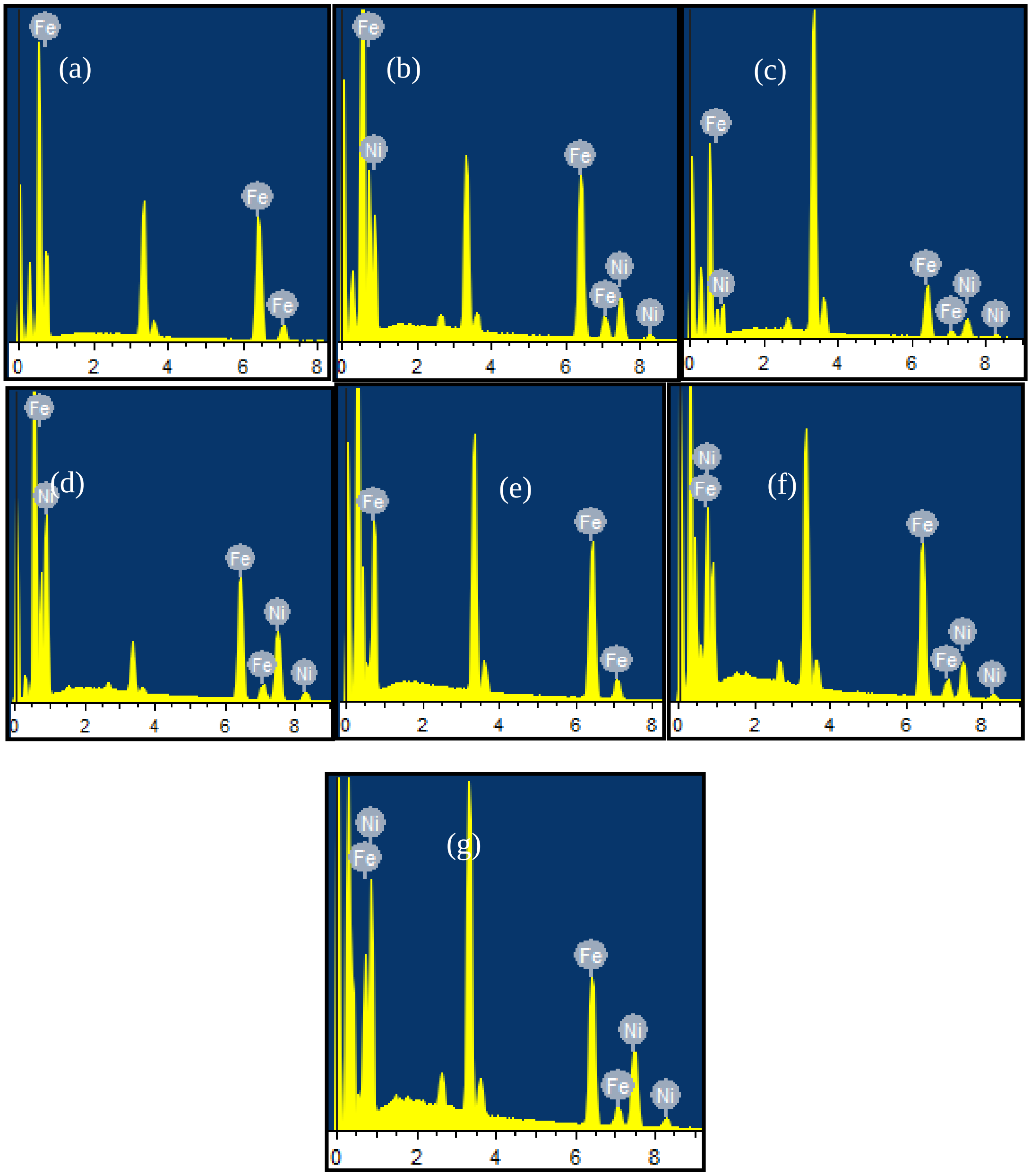

Figure S9. EDS spectra of Fe-PB (a), $NiFe_2$-PB (b), NiFe-PB (c), Ni_2Fe-PB (d), Fe-O (e), $NiFe_2$-O (f), NiFe-O (g).

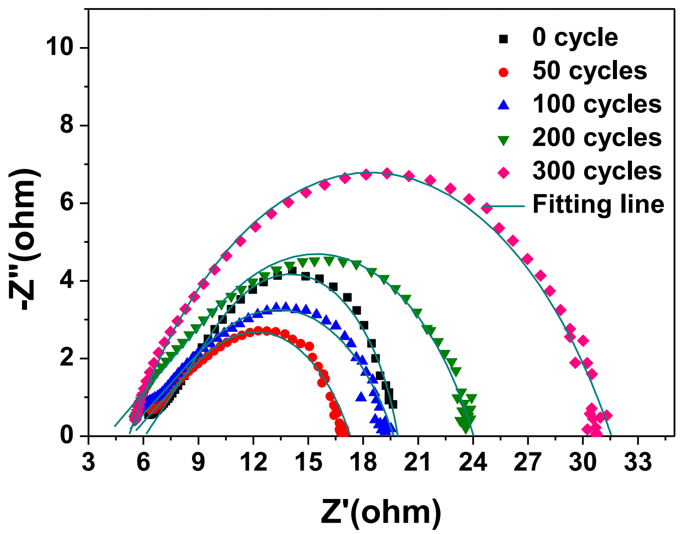

Figure S10. Nyquist plots of Ni_2Fe-O in different cycles.

1.2 Supplementary Tables

Table S1. The nickel and iron contents of the pristine PBAs.

Samples	Elements	Molar ratios (at.%)
Fe-PB	Fe	100
$NiFe_2$-PB	Fe	69.15
	Ni	30.85
NiFe-PB	Fe	49.82
	Ni	50.18
Ni_2Fe-PB	Fe	49.37
	Ni	50.63

Table S2. The nickel and iron contents of the derivatives after calcination at 500 °C.

Samples	Elements	Molar ratios (at.%)
Fe-O	Fe	100
$NiFe_2$-O	Fe	70.72
	Ni	29.28
NiFe-O	Fe	52.44
	Ni	47.56
Ni_2Fe-O	Fe	44.70
	Ni	55.30

Table S3. Comparison of OER properties for different materials.

Materials	η^a (mV)	η^b (mV)	References
Ni_2Fe-O	370	270	in this study
$NiFe_2O_4$ nanopartiles	—	470	Li et al., 2015
$NiFe_2O_4$ nanofibers	—	440	Li et al., 2015
$Fe_{0.5}Ni_{0.5}O_x$	584	—	Jiang et al., 2016
NiO	430	—	Jung et al., 2016
$NiFe_2O_4$	500	—	Jung et al., 2016
Ni-Co mixed oxide porous cubes	430	—	Han et al., 2016
Fe_3Ni_2O	—	270	Chen et al., 2014
NiOH nanoplate	360	270	Yu et al., 2016
Ni-Co mixed oxide cages	380	—	Han et al., 2016

Table S4. Comparison of OER properties for different catalysts in this study.

Samples	η^a@10 mA cm^{-2} (mV)	Tafel slope (mV dec^{-1})	Rct (Ω)
Ni_2Fe-O	370	48	4.1
NiFe-O	410	64	15.8
$NiFe_2$-O	450	77	38.2
Fe-O	540	93	69.8
Ni_2Fe-O600	420	61	27.3
Ni_2Fe-O700	430	69	36.0
